# 

**Published:** 1910-05

**Authors:** 


### CONTENTS.

ORIGINAL COMMUNICATIONS—
Diphtheria and its Relation to the Laboratory. By E. R. Collins, M. D., Atlanta, Ga.. 53
Tenotomy. By Theodore Toepel, M. D., Atlanta, Ga. ... 58
Acute Infantilism. By H. McH. Hull, M. D., Atlanta, Ga. ... 68
Hydrophobia, its Prevalence and Prevention. By Henry R. Slack, M. D., Superintendent of the LaGrange Sanatorium, LaGrange, Ga. ... 74
Radium; its Use in Some Cases. By Theodore Eugene Oertel, M. D., Augusta, Ga.. 76
The Value of Gonococcic Vaccine in the Treatment of Gonorrheal Arthritis. By W. L. Champion, M. D., Atlanta, Ga. ... 83
Report of Surgical Cases. By R. R. Kime, M. D., Atlanta, Ga. ... 85
Penetrating Wounds of the Chest and Abdomen With Report of a Recent Case. By C. C. Abell, M. D., Texarkana, Tex. ... 92
Report of an Unusual Case of Pneumonia. By J. E. Sommerfield, M. D., Atlanta, Ga.. 98
EDITORIALS ... 100
COMMUNICATIONS ... 102
BOOK REVIEWS t ... 106

